# Impact of Comprehensive Health Insurance affiliation on mortality in children under one year: an analysis of the Demographic and Health Survey 2010–2022 in Peru

**DOI:** 10.3389/fpubh.2024.1405244

**Published:** 2025-01-23

**Authors:** Marcos Espinola-Sánchez, Andres Campaña-Acuña, Diego Urrunaga-Pastor, Jorge L. Maguiña, Manuel Jumpa, Oscar Ugarte-Ubillus

**Affiliations:** ^1^Facultad de Ciencias de la Salud, Universidad Privada del Norte, Lima, Peru; ^2^Dirección de Investigación, Escuela Nacional de Salud Pública, Ministerio de Salud, Lima, Peru; ^3^Carrera de Medicina Humana, Facultad de Ciencias de la Salud, Universidad Científica del Sur, Lima, Peru

**Keywords:** Comprehensive Health Insurance, impact assessment, Demographic and Health Survey, Cox model, infant mortality

## Abstract

**Objective:**

To assess the impact of Comprehensive Health Insurance (CHI) coverage on mortality in children under 1 year of age in the Peruvian population from 2010 to 2022. Additionally, the study evaluated how CHI affects infant mortality according to wealth quintiles in the Peruvian population.

**Methods:**

A causal inference analysis with observational data was applied, employing propensity score matching. Participants included children under 1 year of age, surveyed through the National Demographic and Family Health Survey (ENDES) from 2010 to 2022. Variables measured included CHI affiliation and death in children under 1 year. Methods such as inverse probability weighting adjusted by regression, Average Treatment Effect on the Treated (ATET) estimation, and endogeneity test were applied.

**Results:**

The study population consisted of 26,319 children under 1 year, with 11,922 not affiliated with CHI and 14,397 affiliated. The ATET analysis as an exogenous treatment of CHI showed a significant reduction in infant mortality in the overall population of children by 12.6% (95% CI: 12.1 to 13.1%), in the poverty subgroup by 15.6% (95% CI: 14.9 to 16.3%), and in the non-poverty subgroup by 6% (95% CI: 5.5 to 6.4%). However, endogeneity was observed in the ATET for the non-poverty subgroup and for the overall population of children.

**Conclusion:**

CHI affiliation contributes to reducing mortality in children under 1 year in the population with low economic incomes in Peru. However, this relationship is inconclusive for the general population and for the population with medium and high incomes, highlighting the importance of considering socioeconomic, demographic, and other insurance factors.

## Introduction

In Latin America and the Caribbean, health inequalities persist, particularly affecting infant mortality, with more pronounced effects in rural areas and among the poorest quintiles (1, 2). Addressing these disparities is essential for reducing infant mortality rates and achieving sustainable development goals (SDGs) (1). In low- and middle-income countries, it is estimated that 58% of infant deaths could have been prevented without socioeconomic and geographic inequalities (2). Several Latin American countries have adopted universal coverage with multisectoral interventions to improve health and its determinants (3). Providing global and regional infant mortality figures is indicated.

Universal Health Coverage, driven by the WHO, aims to ensure equitable access to quality services without imposing financial burdens (4). National governments and the international community emphasize the importance of strengthening primary care systems and universal health coverage in the context of SDGs and epidemiological changes (5). However, the definition and evaluation of universal health coverage indicators are still unclear in the context of SDGs (4). On the other hand, Latin America exhibits health segregation between social security and Ministry of Health services; although improvements have been made, this system is considered an obstacle to the right to health (6).

In Peru, the implementation of the Comprehensive Health Insurance (CHI) in 2009 was a key step toward universal health coverage (7). Health reform in 2013 expanded this coverage by universalizing CHI and tripling its budget (8). By 2019, 88.2% of the population was affiliated with health insurance, predominantly CHI (52%) covering mainly the impoverished population; secondly, Social Health Insurance (30%) accessed through monthly employer payments or direct payments by the affiliate; and thirdly, private insurance and health services (6%) (9). Despite these achievements, public health funding remains insufficient (9, 10). It is essential to highlight that health policies aim to improve health and promote social well-being by reducing mortality, morbidity, and disability in the context (11).

Although global child mortality has decreased, disparities persist, and children in locations with low sociodemographic indices experience the majority of deaths and disability burden (12). In Latin America and the Caribbean, there have been advancements in health intervention coverage and the reduction of inequalities in infant mortality; however, gaps persist, especially in Bolivia, Guatemala, Haiti, Nicaragua, and Peru (13). In Peru, infant mortality has decreased due to factors such as economic growth, improvements in social determinants, and increased health spending, although socioeconomic and regional inequalities persist (14).

Despite advances in health coverage in low- and middle-income countries, evidence on the direct impact of public health insurance on infant mortality remains limited. Previous studies have emphasized the need for research that not only evaluates the average effect of health insurance coverage but also examines how this impact varies across different socioeconomic groups, particularly in contexts of high inequality. While increased health insurance coverage generally appears to improve access to healthcare facilities, provide financial protection, and enhance health outcomes, findings are not entirely consistent. Understanding the factors that drive these differences in the outcomes of insurance reforms is crucial for informing future implementations of publicly funded health insurance and achieving the broader goal of universal health coverage (15).

This study is distinguished by its detailed analysis of how affiliation with CHI in Peru has affected mortality in children under 1 year of age from 2010 to 2022, specifically evaluating how this effect varies according to wealth quintiles. The findings of this research are expected to provide valuable insights into the equity and effectiveness of health policies in the region, potentially informing and supporting future reforms in universal health coverage.

## Methods

### Study design

An observational cross-sectional analytical study was conducted using population-based data from the National Demographic and Family Health Surveys (ENDES) in Peru, spanning from 2010 to 2022. This study adheres to the STROBE guidelines for reporting observational research in the health field (16). A causal inference analysis with observational data was applied, employing propensity score matching.

### Data source

ENDES is conducted by the National Institute of Statistics and Informatics (INEI) of Peru, representing a nationally representative survey in all 24 regions of Peru and the Constitutional Province of Callao, within the framework of the World Program of Demographic and Health Surveys (17). ENDES provides official and up-to-date information on fertility determinants, mortality, and health in Peru (17). It employs a standardized approach to ensure data comparability on health across different countries (18). In Peru, ENDES has been conducted annually since 2004 (19) and is currently available as publicly accessible databases provided by the Peruvian government through the “Microdatos” section of the official INEI website, available at https://proyectos.inei.gob.pe/microdatos/.

### Population and sample

The population included infants under 1 year of age in Peru. All infants under 1 year of age in Peru and their mothers (women in their fertile stage aged 15–49) who participated in ENDES were included. Exclusion criteria included ENDES surveys without information on CHI affiliation for the child, incomplete questionnaire data categorized by the ENDES database, and women of foreign origin. Therefore, the analysis included all infants under 1 year and their mothers who participated in ENDES from 2010 to 2022 and met the inclusion and exclusion criteria.

### Sampling design

ENDES employs a cross-sectional design to obtain participant data, using a two-stage probability sampling. The first stage involves the random selection of geographical areas or clusters, and the second stage involves the random selection of households within the selected clusters. Data collected by ENDES are through interviews using pre-tested questionnaires covering a wide range of health and demographic topics. The sampling methodology has been documented for different years, detailed in methodological reports and technical sheets for each ENDES (20), available on the official INEI website https://proyectos.inei.gob.pe/endes/documentos.asp.

### Variables

The exposure variable in this study was the child’s affiliation with CHI, recorded dichotomously as being affiliated or not affiliated with CHI. The outcome variable was infant mortality under 1 year, measured dichotomously as present or absent. Additionally, the time to the infant’s death or survival was recorded as the number of months until death or age in months if still alive at the time of the survey. Other covariates related to the mother were collected, including the mother’s age at the time of the child’s birth, mother’s educational level, mother’s literacy, partner’s educational level, marital status dichotomized as married or cohabiting and other marital statuses, and parity measured as the number of live births the mother had before the current birth.

Information on prenatal care was also collected, dichotomously measured as having or not having at least six prenatal check-up visits by a healthcare professional during pregnancy; interval to the previous birth, measured as the time in months between the previous birth and the last reported birth; institutional childbirth care, dichotomously measured if the childbirth was assisted or not in a healthcare facility; and the child’s gender, dichotomously measured as male or female.

Contextual information such as wealth index defining the household’s economic position in wealth quintiles (very poor, poor, medium, rich, and very rich); mother’s place of residence at the time of the survey, categorized as Urban or Rural; mother’s geographic region at the time of the survey, according to Peru’s administrative divisions; year of the survey conducted, representing the year of the ENDES database, was also collected.

These covariates were considered to adjust the analyses for possible confounding factors in the relationship between the child’s affiliation with CHI and infant mortality under 1 year.

### Data analysis

For categorical variables, descriptive results were presented using absolute and relative frequencies. Quantitative variables were described using means and standard deviations or medians and interquartile ranges, depending on the variable’s distribution. Tables were used to compare summary measures of different variables collected based on whether the child had CHI affiliation.

For the main analysis, simple propensity scores were estimated, adjusting for covariates (mother’s place of residence at the time of the survey, categorized as Urban or Rural; the mother’s geographic region at the time of the survey, according to Peru’s administrative divisions, and the year the survey was conducted), using the inverse probability weighting regression adjustment (IPWRA) technique. This analysis generated a pseudo-population where the child’s CHI affiliation is independent of observed covariates. Common support of propensity scores between treated (CHI-affiliated children) and control groups (non-CHI-affiliated children) was assessed through overlap graphs of the propensity score distribution before and after weighting to validate the application of inverse probability of treatment weighting (IPTW). Subsequently, IPWRA weights were applied in a regression model to estimate the causal effect of the child’s CHI affiliation on infant mortality.

Sampling weights provided by ENDES were also considered in the analyses to ensure that the estimates are nationally representative of Peru. To achieve this, composite weights were obtained by combining propensity score weights and ENDES sampling weights. Once the composite propensity and sampling weights were obtained, they were applied in evaluating the effect of CHI affiliation on infant mortality.

The estimated effect size was the Average Treatment Effect on the Treated (ATET), used to estimate the treatment effect (CHI affiliation) on the outcome of death (reported in terms of difference in proportions) using the IPWRA technique. For the outcome of months elapsed until death (reported in terms of difference in means), a Cox regression with censoring and matching was employed. The effect of CHI affiliation on infant mortality was estimated for the population of children under 1 year overall, and subgroup analyses were performed based on wealth quintiles, grouping into a population with poverty (quintile “very poor” and “poor”) and a population without poverty (quintile “medium,” “rich,” and “very rich”).

Subsequently, endogeneity tests were conducted, as endogeneity can significantly alter ATET values and their confidence intervals. The endogeneity test was employed, with the null hypothesis indicating non-endogeneity in treatment and the alternative suggesting endogeneity in treatment, necessitating a change in the estimation technique known as endogenous treatment regressions.

All data processing and analysis were performed using Stata version 16.

### Ethical considerations

This study involved secondary analysis of open ENDES data, which consists of anonymized and publicly accessible databases. The research protocol was submitted to the institutional ethics committee of the National School of Public Health for evaluation. Data handling was anonymized due to the nature of the information source and used only for the purposes of this research.

## Results

### Population characteristics

**Figure 1:**
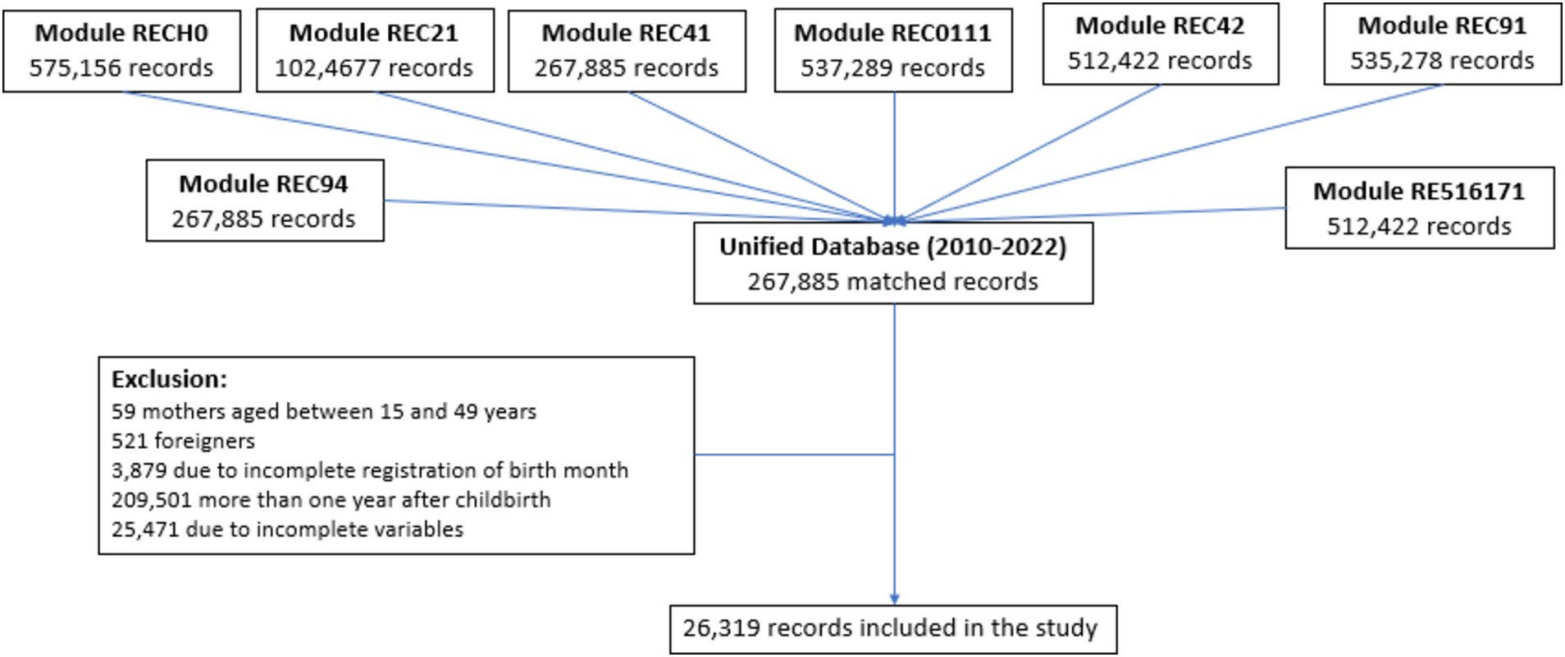
Flowchart of the selection of the study sample (2010–2022). RECH0, Household Characteristics; REC21, Birth History – Knowledge Method Table; REC41, Pregnancy, Childbirth, Postpartum, and Breastfeeding – Care during and after Pregnancy; REC0111, Basic Data of Women of Childbearing Age – Residence and Demographic Characteristics; REC42, Immunization and Health; REC91, Basic Data of Women of Childbearing Age – Reproductive Context Information and Media; REC94, Pregnancy, Childbirth, Postpartum, and Breastfeeding – Variables on Complications and Nutrition; RE516171, Nuptiality – Fertility – Spouse and Woman.

The study population comprised 26,319 children, of whom 11,922 were not affiliated with CHI, and 14,397 were affiliated (Figure 1).

Children affiliated with CHI exhibited differences compared to those without CHI affiliation; they had a lower frequency of death (0.8% according to weighted percentage), a higher median age (median of 7 months, with a minimum of 4 months and a maximum of 10 months), a higher frequency of at least 6 prenatal check-ups (85.1% according to weighted percentage), predominantly resided in rural areas (38.3% according to weighted percentage), lower wealth index (36.4% for “very poor” quintile and 35% “poor” quintile according to weighted percentages), lower frequency of higher university education in mothers (5.3% according to weighted percentage), and in partners (5% according to weighted percentage) (Table 1).

**Table 1:** Population characteristics according to child affiliation status to the Comprehensive Health Insurance (CHI) using continuous ENDES from 2010 to 2022.

Variables	Child’s CHI affiliation			*p* value
		No (*n* = 11,922) *n* (%)	Yes (*n* = 14,397) *n* (%)	
Child’s death	No	10,538 (89.5%)	14,270 (99.2%)	<0.001
	Yes	1,384 (10.5%)	127 (0.8%)	
Months to the child’s death		5.5 (2.0–9.0)	7.0 (4.0–10.0)	<0.001
Minimum 6 prenatal check-ups	No	2,332 (20.1%)	2,228 (14.9%)	<0.001
	Yes	8,894 (79.9%)	11,976 (85.1%)		
Institutional delivery	No	1,593 (10.9%)	1,291 (6.2%)	<0.001
	Yes	10,329 (89.1%)	13,106 (93.8%)		
Year	2010	601 (3.4%)	579 (1.8%)	
	2011	713 (3.7%)	414 (1.3%)	
	2012	950 (4.8%)	165 (0.5%)	
	2013	801 (4.2%)	172 (0.5%)	
	2014	793 (3.4%)	164 (0.4%)	
	2015	1,684 (15.3%)	478 (2.7%)	
	2016	1,120 (9.5%)	1,675 (9.7%)	
	2017	1,142 (12.0%)	1,775 (13.2%)		
	2018	1,117 (11.2%)	1,826 (13.4%)		
	2019	980 (10.4%)	1,838 (13.4%)		
	2020	403 (3.4%)	894 (5.8%)	
	2021	807 (9.2%)	2,136 (18.0%)		
	2022	811 (9.4%)	2,281 (19.3%)		
Residence	Urban	8,590 (75.0%)	8,328 (61.7%)	<0.001
	Rural	3,332 (25.0%)	6,069 (38.3%)		
Literacy	Can read part of sentences	752 (4.8%)	982 (5.4%)	0.23
	Can read complete sentences	306 (2.4%)	534 (3.3%)	
	Does not require language cards	10,864 (92.8%)	12,881 (91.4%)		
Region	Amazonas	426 (4.8%)	669 (6.0%)	<0.001
	Ancash	399 (1.6%)	517 (1.8%)	
	Apurímac	224 (2.2%)	627 (5.1%)	
	Arequipa	491 (2.1%)	318 (1.1%)	
	Ayacucho	269 (2.3%)	709 (5.0%)	
	Cajamarca	237 (0.7%)	648 (1.7%)	
	Callao	509 (2.9%)	356 (1.6%)	
	Cusco	311 (1.2%)	591 (1.8%)	
	Huancavelica	223 (2.1%)	608 (5.4%)	
	Huánuco	297 (1.9%)	739 (4.0%)	
	Ica	595 (3.3%)	390 (1.8%)	
	Junín	455 (1.8%)	496 (1.6%)	
	La Libertad	517 (1.3%)	440 (0.9%)	
	Lambayeque	470 (2.1%)	525 (1.8%)	
	Lima	1,506 (3.8%)	1,172 (3.1%)	
	Loreto	691 (2.7%)	773 (2.5%)	
	Madre de Dios	533 (16.0%)	575 (15.1%)	
	Moquegua	402 (12.0%)	326 (8.1%)	
	Pasco	473 (8.4%)	499 (7.2%)	
	Piura	608 (1.6%)	596 (1.2%)	
	Puno	286 (1.1%)	437 (1.5%)	
	San Martín	423 (2.3%)	735 (3.1%)	
	Tacna	359 (5.5%)	414 (5.6%)	
	Tumbes	494 (9.5%)	552 (8.6%)	
	Ucayali	724 (6.5%)	685 (4.7%)	
Wealth index	Very poor	2,755 (19.8%)	5,701 (36.4%)	<0.001
	Poor	2,632 (24.5%)	4,549 (35.0%)		
	Middle	2,472 (22.0%)	2,583 (18.4%)		
	Rich	2,212 (19.6%)	1,183 (8.0%)	
	Very rich	1,851 (14.1%)	381 (2.2%)	
Maternal age	15–19	232 (1.7%)	362 (2.4%)	0.072
	20–24	1,638 (13.0%)	2,500 (16.4%)		
	25–29	2,942 (24.6%)	3,840 (26.3%)		
	30–34	3,380 (29.7%)	3,768 (26.9%)		
	35–39	2,531 (21.2%)	2,702 (19.5%)		
	40–44	1,084 (9.0%)	1,103 (7.7%)	
	45–49	115 (0.9%)	122 (0.8%)	
Educational level	None, preschool	259 (1.5%)	351 (1.9%)	<0.001
	Primary	2,646 (18.2%)	4,432 (25.8%)		
	Secondary	4,792 (39.4%)	7,418 (54.4%)		
	Non-university higher education	2,307 (21.8%)	1,567 (12.6%)		
	University degree	1,728 (17.2%)	622 (5.3%)	
	Postgraduate	190 (1.9%)	7 (0.1%)	
Partner’s educational level	None, preschool	117 (0.9%)	153 (1.0%)	<0.001
	Primary	1,992 (13.0%)	3,367 (18.7%)		
	Secondary	5,137 (42.1%)	8,311 (59.0%)		
	Non-university higher education	2,429 (22.3%)	1,633 (13.5%)		
	University degree	649 (10.7%)	1,037 (5.0%)	
	Postgraduate	90 (1.5%)	35 (0.2%)	
	Does not know	12 (0.2%)	75 (0.4%)	
Interval between previous births		53.5 (32.0–86.0)	59.0 (36.0–92.0)	<0.001
Married/cohabiting	No	844 (7.3%)	1,138 (8.5%)	0.035
	Yes	11,078 (92.7%)	13,259 (91.5%)		
Parity	Single birth	11,780 (98.8%)	14,249 (98.9%)	0.57
	Multiple births (1st birth)	142 (1.2%)	148 (1.1%)	
Child’s gender	Male	6,134 (51.2%)	7,303 (50.5%)	0.25
	Female	5,788 (48.8%)	7,094 (49.5%)		

*n represents the unweighted absolute frequency; % represents the weighted column percentage. Chi-square test is shown for categorical variables, and U-Mann Whitney test is used for count variables.*

The affiliation to the SIS of children between 2010 and 2022 shows an increasing trend. Initially, in 2010, less than half of the children (49.1%) were affiliated, but starting in 2016, affiliation increased, exceeding 60% from 2017 onwards and reaching its highest level in 2022 with 73.8%. Throughout the years, the total percentage of children affiliated with SIS was 54.7%. Regarding child deaths between 2010 and 2022, the annual percentage of deaths has been relatively low, ranging from 3.8 to 11.5%. The overall trend shows a progressive decrease in deaths starting in 2015, with a total percentage of deaths of 5.7% during the period. A detailed description of this information can be found in Supplementary material.

### Propensity score estimation, weights, and impact analysis assumptions

**Figure 2:**
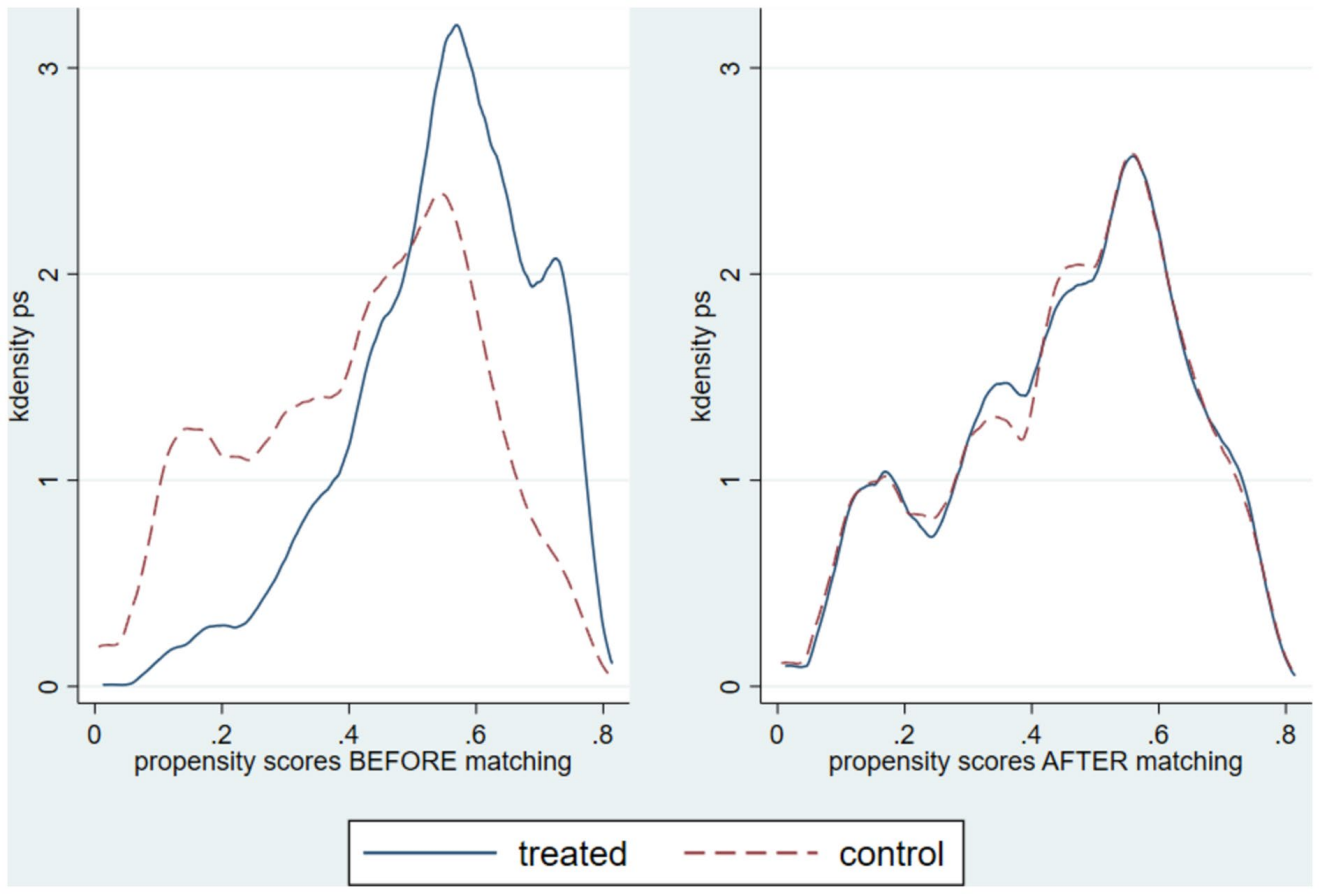
Distribution of propensity scores before and after weighting by IPTW, evaluating the common support between CHI-affiliated and non-affiliated individuals by year.

**Figure 3:**
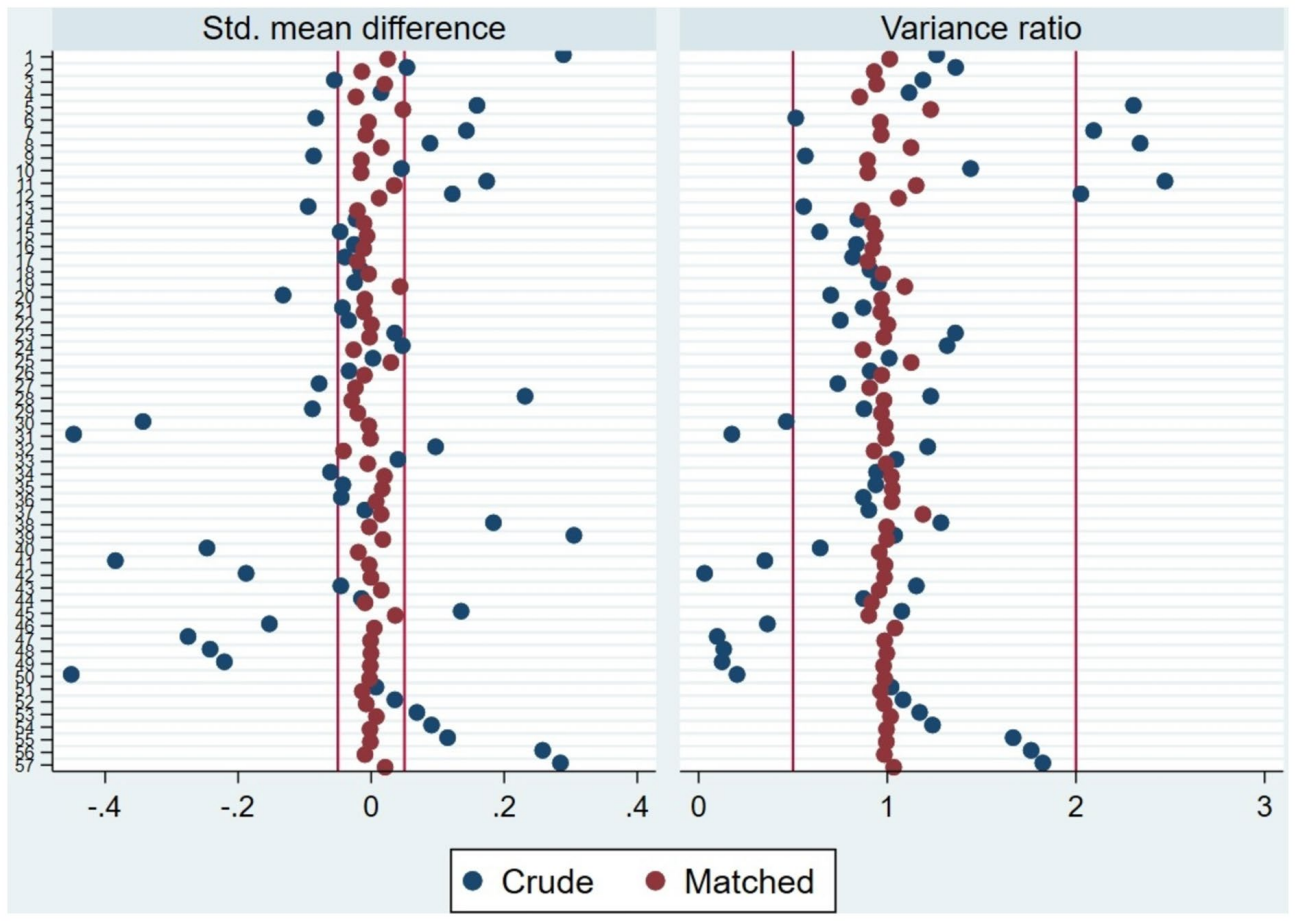
Graph of covariate balance before and after weighting. 1: rural area; 2: can only read parts of sentences; 3: reads complete sentences; 4: Áncash; 5: Apurímac; 6: Arequipa; 7: Ayacucho; 8: Cajamarca; 9: Callao; 10: Cusco; 11: Huancavelica; 12: Huánuco; 13: Ica; 14: Junín; 15: La Libertad; 16: Lambayeque; 17: Lima; 18: Loreto; 19: Madre de Dios; 20: Moquegua; 21: Pasco; 22: Piura; 23: Puno; 24: San Martín; 25: Tacna; 26: Tumbes; 27: Ucayali; 28: Poor; 29: Middle; 30: Rich; 31: Very rich; 32: 20–24 years; 33: 25–29 years; 34: 30–34 years; 35: 35–39 years; 36: 40–44 years; 37: 45–49 years; 38: Primary; 39: Secondary; 40: Non-university higher education; 41: University higher education; 42: Postgraduate; 43: married/cohabitant; 44: Multiple birth (1st of birth); 45: interval with the previous birth; 46: year of interview 2011; 47: year of interview 2012; 48: year of interview 2013; 49: year of interview 2014; 50: year of interview 2015; 51: year of interview 2016; 52: year of interview 2017; 53: year of interview 2018; 54: year of interview 2019; 55: year of interview 2020; 56: year of interview 2021; 57: year of interview 2022.

To estimate the effect of the child’s affiliation with CHI on death in children under 1 year, the inverse probability of treatment weighting (IPTW) method was applied. The distribution of propensity scores before and after weighting demonstrated the validity of IPTW application. Although there was common support between treated (CHI-affiliated children) and control (non-CHI-affiliated children) groups before weighting, the distributions were different. After applying IPTW, the distributions in all study years aligned notably, indicating adequate balance to ensure unbiased estimates due to the positivity assumption (Figures 2, 3).

### Estimated effect of CHI affiliation on infant mortality under 1 year

#### ATET calculated for exogenous treatment

ATET was estimated for the outcome of death (reported as a difference in proportions) and for the outcome of months until death (reported as a difference in means). These effects were estimated for the total population of children under 1 year and analyzed by subgroups based on wealth quintiles, grouping into a population with poverty (“very poor” and “poor” quintiles) and a population without poverty (“medium,” “rich,” and “very rich” quintiles).

In the estimation of ATET considering the total group of children under 1 year, a significant effect estimate of the child’s affiliation with CHI on death was obtained, showing a reduction in the proportion of deaths by 12.6% (95% CI: 12.1 to 13.1%). When analyzed in subgroups, a reduction in the proportion of deaths was observed by 15.6% (95% CI: 14.9 to 16.3%) in the poverty group, while a reduction of 6% (95% CI: 5.5 to 6.4%) was observed in the non-poverty group. There was no significant evidence for the effect on months until death (Table 2).

**Table 2:** Estimated ATET using continuous ENDES from 2010 to 2022.

Type	Variable	ATET	Lower CI	Upper CI
Total	Mortality	−0.126	−0.131	−0.121
	Months to mortality	0.320	−0.810	1.450
Poor	Mortality	−0.156	−0.163	−0.149
	Months to mortality	−0.148	−1.579	1.283
Non-poor	Mortality	−0.060	−0.064	−0.055
	Months to mortality	–	–	–

#### ATET calculated for endogenous treatment

ATET was estimated for the outcome of death (reported as a difference in proportions) and for the outcome of months until death (reported as a difference in means). An endogeneity test was applied for the total group and analyzed by subgroups based on wealth quintiles, grouping into a population with poverty (“very poor” and “poor” quintiles) and a population without poverty (“medium,” “rich,” and “very rich” quintiles).

Regarding the ATET estimate reported in the difference in proportions, no endogeneity was observed in the analysis of the CHI effect on mortality in the poverty subgroup (*p* = 0.568), unlike the total group and the non-poverty subgroup (*p* < 0.001). The ATET estimate reported in differences in means did show endogeneity in the analysis of the CHI effect on mortality in the total group and the poverty and non-poverty subgroups (*p* < 0.001). A reduction in the proportion of deaths by 15.7% (95% CI: 14.9 to 16.5%) was observed in the poverty group. However, endogeneity tests in treatment indicated that the ATET value obtained in the total group and the non-poverty group could be significantly altered (Table 3).

**Table 3:** Endogeneity test and calculated ATET according to endogenous treatment.

Type	Variable	Endogeneity test	ATET	Lower CI	Upper CI
Total	Mortality	<0.001	0.221	0.219	0.224
	Months to mortality	<0.001	−0.136	−0.245	−0.028
Poor	Mortality	0.568	−0.157	−0.165	−0.149
	Months to mortality	<0.001	0.989	0.740	1.239
Non-poor	Mortality	<0.001	0.200	0.197	0.203
	Months to mortality	<0.001	−2.356	−2.778	−1.934

## Discussion

This study aimed to investigate the impact of affiliation with the CHI on infant mortality in Peru. Disparities were observed in various factors between CHI-affiliated and non-affiliated children, such as prenatal check-ups, institutional childbirth, residence, region, wealth index, maternal marital status, and educational levels of both the mother and her partner. Addressing these differences through propensity scores revealed a 15% reduction in mortality among CHI-affiliated children, particularly within the impoverished group. However, in other analysis groups, the impacts of CHI were inconsistent in endogeneity tests, both for the total group of children and the subgroup without poverty.

The findings demonstrated an association between CHI affiliation and infant mortality in Peru, indicating a lower overall percentage of deaths among CHI-affiliated individuals compared to non-affiliated ones. This finding is consistent with previous research emphasizing the role of health coverage in reducing infant mortality (21, 22). However, the study also uncovered inequalities in maternal prenatal care compliance and socioeconomic disparities between affiliated and non-affiliated individuals, suggesting that these inequalities could influence the infant mortality rate (2).

In this study, infants under 1 year affiliated with CHI more frequently came from mothers who adhered to prenatal check-ups. This evidence aligns with previous research in Peru, highlighting the association of CHI with more consistent maternal prenatal care (23). Adequate prenatal monitoring plays a crucial role in preventing complications that could lead to fatal outcomes for both mother and child (24). Additionally, the number of prenatal check-ups has been identified as a significant predictive factor for neonatal mortality (25). It is estimated that increased health coverage in the prenatal and postnatal stages could prevent 71% of neonatal deaths by 2025 (21).

In Peru, CHI coverage has gradually increased, reaching 52% of the population in 2019, while 30% are covered by Social Health Insurance, and 6% have private or other health insurance (9). It has been reported that the characteristics of the population differ according to the type of insurance (26). These differences align with the findings of the present study, where CHI-affiliated infants more frequently come from rural areas, have higher poverty indices, and to a lesser extent, have higher education levels. Addressing these socioeconomic and geographic inequalities is crucial, as their elimination could prevent up to 58% of child deaths in developing countries (2).

In this study, we applied propensity scores adjusted for covariables to assess the impact of CHI affiliation on infant mortality, while also considering the weighting of the complex ENDES sample to make inferences at the national level. The propensity score-based method has become increasingly common in surveys with complex population designs, allowing for the estimation of exposure or intervention effects and mitigating the confusion caused by other characteristics or factors in the population (27).

The use of propensity scores revealed a 15% reduction in infant mortality among CHI-affiliated children, particularly in the low-income population. However, this reduction was not consistent for children without poverty, nor in the overall analysis of children under 1 year. This inconsistency may be explained by differences in population characteristics according to insurance type (26). In low-income households, obtaining insurance was related to the gradual implementation of CHI; in contrast, in non-poverty groups, having some health insurance could be linked to Social Health Insurance, CHI, or private insurance (9). However, the ENDES data source records the child’s affiliation as CHI-affiliated or not since 2010, without differentiating other types of health insurance. In this context, our analysis detected endogeneity problems when measuring the impact of CHI on children without poverty and the total population. Therefore, our findings support the appropriate interpretation of the impact of CHI, focusing on the low-income population rather than the entirety.

Furthermore, our findings underscore the importance of considering socioeconomic inequalities when measuring the impact of health policies on infant mortality. Previous research also highlights that these socioeconomic and geographic inequalities determine different infant mortality rates within the same country (2). Similarly, studies on CHI in Peru indicate issues regarding the sufficiency and equity of the system (28).

On the other hand, it is relevant to highlight that immunization has contributed to the reduction of infant mortality, thanks to government efforts focused on preventing diseases such as pneumonia, diarrhea, and malaria (29). However, in the context of fragmented programs, immunization might not show a significant impact on infant mortality compared to internal investment in healthcare (30). In the specific case of Peru, both CHI-affiliated and non-affiliated children are beneficiaries of the national vaccination program.

One of the main limitations of our study is its cross-sectional design, preventing the establishment of definitive causal relationships between CHI affiliation and infant outcomes. Additionally, despite controlling for multiple covariables, there always remains the possibility of confusion by unmeasured variables that could influence the results. Another limitation was the inability to conduct a subset analysis by time due to the imbalance in the number of infant death events per year, which could have provided more detailed information on time-dependent differences between children affiliated and not affiliated with CHI. Although the years were included as dichotomous variables in the propensity score analysis to mitigate this potential bias, we cannot rule out the possibility that time-related factors not measurable in ENDES, such as the expansion of CHI or changes in public health policies, may have influenced the observed results. Future studies with more balanced data could explore these temporal effects and provide a more comprehensive view of the impact of CHI on infant mortality. Finally, another limitation of the study is the exclusion of children with incomplete registration information, which could influence the results. Despite these limitations, this study provides a valuable first approach to a little-evaluated issue in the healthcare system of populations like ours and lays the groundwork for further studies.

Despite the aforementioned limitations, the evidence suggests that affiliation with CHI can have a positive impact on infant health. Given that CHI covers more than half of the Peruvian population, even slight variations in proportions yield significant effects at the population level. These findings hold potential value for public health policymakers when contemplating the expansion, modification, or reinforcement of CHI. It is crucial to persist in evaluating CHI to optimize its advantages for infant health while acknowledging and addressing the constraints posed by socioeconomic inequalities within the population.

## Conclusion

The study yielded significant findings that enhance our comprehension of the impact of CHI affiliation on infant mortality in populations akin to the Peruvian context. Children under 1 year affiliated with CHI exhibited substantial socioeconomic disparities compared to their counterparts. Within this framework, a notable reduction in mortality rates was observed among children associated with CHI, particularly pronounced in impoverished households.

However, the inconsistency in the observed effects of CHI on the overall population of children under 1 year, as well as on children without poverty, underscores the inherent complexity of this relationship within these subgroups, given the presence of alternative insurance options and socioeconomic disparities. This underscores the necessity of considering the nuances of different population segments when evaluating the impact of CHI affiliation on infant mortality.

Understanding the socioeconomic landscape of the study population is crucial for a comprehensive grasp of the effects of CHI affiliation and for formulating more targeted and equitable strategies in the realm of child health in Peru. These findings serve as a foundation for further research and policy analyses aimed at more effectively addressing challenges related to child health in Peru.

## Data Availability

The original contributions presented in the study are included in the article/Supplementary material, further inquiries can be directed to the corresponding author.
