# Supplementary material for: Impact of Comprehensive Health Insurance affiliation on mortality in children under one year: an analysis of the Demographic and Health Survey 2010–2022 in Peru
Source: Front Public Health. 2025 Jan 23;12:1405244. doi: 10.3389/fpubh.2024.1405244 (PMC11798800; doi:10.3389/fpubh.2024.1405244)

**Supplementary material**

**Supplementary table 1. Annual distribution of children affiliated with CHI from 2010 to 2022.**

| Year | CHI | | |
| --- | --- | --- | --- |
|  | No (n, %) | Yes (n, %) | Total (n, %) |
| 2010 | 601 | 579 | 1180 |
|  | 50.93 | 49.07 | 100.00 |
| 2011 | 713 | 414 | 1127 |
|  | 63.27 | 36.73 | 100.00 |
| 2012 | 950 | 165 | 1115 |
|  | 85.20 | 14.80 | 100.00 |
| 2013 | 801 | 172 | 973 |
|  | 82.32 | 17.68 | 100.00 |
| 2014 | 793 | 164 | 957 |
|  | 82.86 | 17.14 | 100.00 |
| 2015 | 1684 | 478 | 2162 |
|  | 77.89 | 22.11 | 100.00 |
| 2016 | 1120 | 1675 | 2795 |
|  | 40.07 | 59.93 | 100.00 |
| 2017 | 1142 | 1775 | 2917 |
|  | 39.15 | 60.85 | 100.00 |
| 2018 | 1117 | 1826 | 2943 |
|  | 37.95 | 62.05 | 100.00 |
| 2019 | 980 | 1838 | 2818 |
|  | 34.78 | 65.22 | 100.00 |
| 2020 | 403 | 894 | 1297 |
|  | 31.07 | 68.93 | 100.00 |
| 2021 | 807 | 2136 | 2943 |
|  | 27.42 | 72.58 | 100.00 |
| 2022 | 811 | 2281 | 3092 |
|  | 26.23 | 73.77 | 100.00 |
| Total | 11922 | 14397 | 26319 |
|  | 45.30 | 54.70 | 100.00 |
|  | | | |

**Supplementary table 2. Annual distribution of child deaths from 2010 to 2022.**

| Year | Child deaths | | |
| --- | --- | --- | --- |
|  | No (n, %) | Yes (n, %) | Total (n, %) |
| 2010 | 1071 | 109 | 1180 |
|  | 90.76 | 9.24 | 100.00 |
| 2011 | 1013 | 114 | 1127 |
|  | 89.88 | 10.12 | 100.00 |
| 2012 | 1009 | 106 | 1115 |
|  | 90.49 | 9.51 | 100.00 |
| 2013 | 870 | 103 | 973 |
|  | 89.41 | 10.59 | 100.00 |
| 2014 | 847 | 110 | 957 |
|  | 88.51 | 11.49 | 100.00 |
| 2015 | 2007 | 155 | 2162 |
|  | 92.83 | 7.17 | 100.00 |
| 2016 | 2657 | 138 | 2795 |
|  | 95.06 | 4.94 | 100.00 |
| 2017 | 2784 | 133 | 2917 |
|  | 95.44 | 4.56 | 100.00 |
| 2018 | 2829 | 114 | 2943 |
|  | 96.13 | 3.87 | 100.00 |
| 2019 | 2675 | 143 | 2818 |
|  | 94.93 | 5.07 | 100.00 |
| 2020 | 1247 | 50 | 1297 |
|  | 96.14 | 3.86 | 100.00 |
| 2021 | 2832 | 111 | 2943 |
|  | 96.23 | 3.77 | 100.00 |
| 2022 | 2967 | 125 | 3092 |
|  | 95.96 | 4.04 | 100.00 |
| Total | 24808 | 1511 | 26319 |
|  | 94.26 | 5.74 | 100.00 |
|  | | | |

**Supplementary figure 1. Trend of child death percentages and SIS affiliation from 2010 to 2022.**
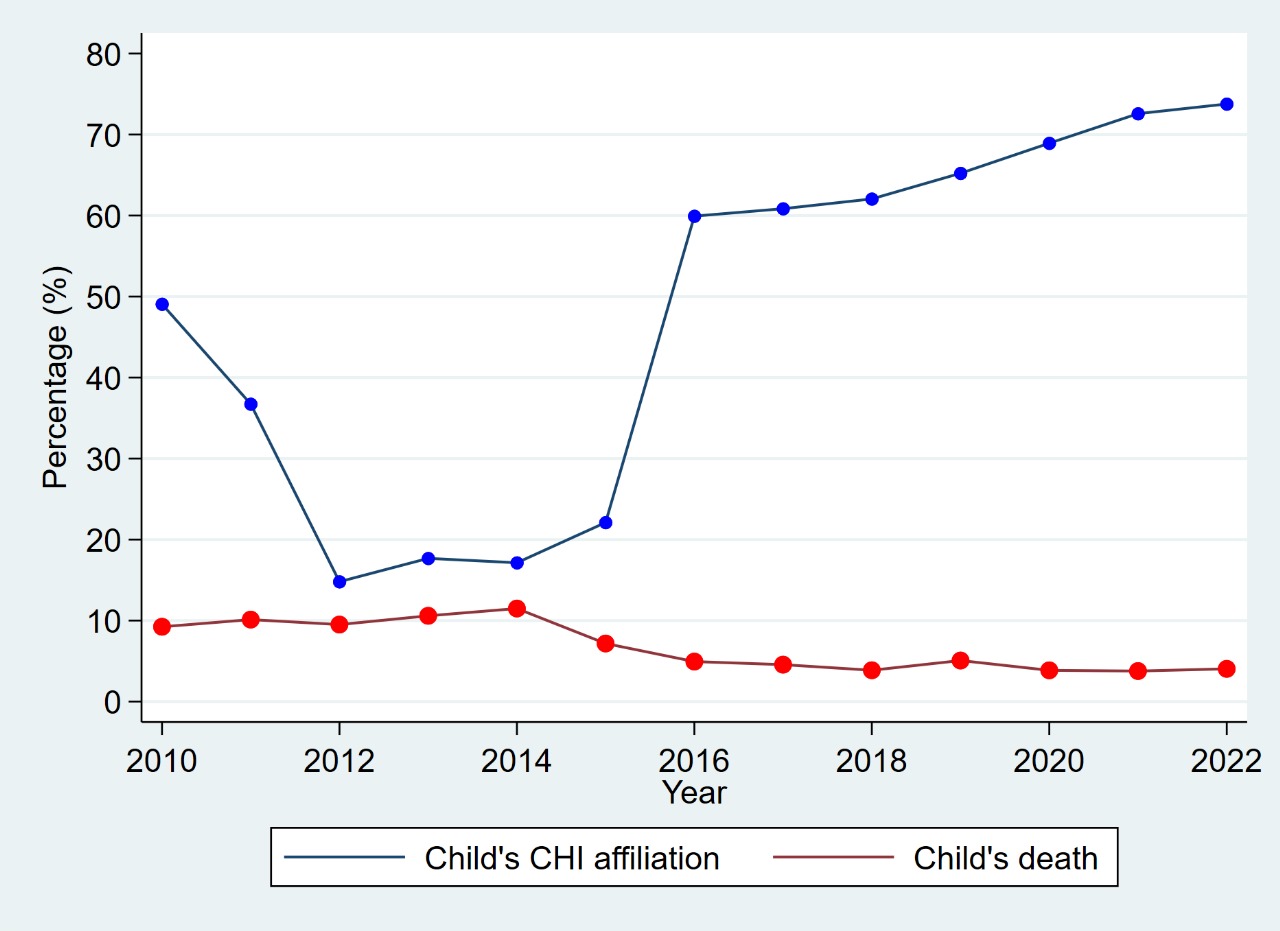

Supplement: Supplementary file 1 [file Table_1.docx]
